# Impact of Awareness, Motivation, and Willingness on the Perception of Age as a Barrier in Adult Orthodontic Treatment: A Questionnaire-Based Study

**DOI:** 10.7759/cureus.94105

**Published:** 2025-10-08

**Authors:** Vikhyathi Dayanand, Rajesh RNG, Rony Kondody, Anushree MK, Roopa K

**Affiliations:** 1 Orthodontics and Dentofacial Orthopedics, Sri Rajiv Gandhi College of Dental Sciences and Hospital, Bengaluru, IND

**Keywords:** adult orthodontics, awareness, motivation, survey, willingness

## Abstract

Background and aim

Orthodontic treatment has traditionally been associated with children and adolescents, as growth potential and skeletal adaptability were believed to optimize treatment outcomes. Adult orthodontics, however, was often overlooked due to concerns about aesthetics, treatment duration, and limited awareness. With the advent of advanced appliances such as ceramic brackets, lingual braces, and clear aligners, alongside a growing emphasis on aesthetics and self-image, orthodontic care has become increasingly relevant for adults. Despite these advancements, perceptions of age as a barrier to treatment persist, highlighting the need to evaluate awareness, motivation, and willingness among adults seeking orthodontic care. This study aimed to assess how awareness, motivation, and willingness influence adult patients’ perceptions of age as a barrier to orthodontic treatment through a questionnaire-based survey.

Objectives

The objectives of the study were to determine whether varying levels of awareness about modern orthodontic treatment options affect perceptions of age as a limiting factor, to analyze how different types of motivation relate to the perceived influence of age on treatment decisions, and to evaluate the role of willingness to undergo orthodontic treatment in mitigating the perception of age as a barrier.

Materials and methods

A quantitative, cross-sectional study was conducted to explore adult perceptions of age as a barrier to orthodontic treatment. The structured questionnaire was validated through expert review, achieving adequate content validity. A total of 118 adult participants, divided into two age groups (below and above 40 years), completed the online questionnaire distributed via convenience sampling. Data were systematically cleaned and analyzed using IBM SPSS Statistics for Windows, Version 21.0 (Released 2012; IBM Corp., Armonk, NY, USA). Descriptive statistics summarized participant responses, and chi-square tests examined associations between categorical variables, with significance set at p < 0.05. Ethical approval was obtained from the Institutional Ethics Committee.

Results

Among 118 participants, 83 (56.8%) were over 40 years of age and 63 (43.2%) were under 40; 89 (61%) were male and 57 (39%) were female. Forty-nine (96.1%) participants under 40 years were aware of an orthodontist compared with 57 (85.1%) of those over 40 years. Age was perceived as a barrier by 51 (76.1%) older adults compared with 29 (56.8%) younger adults. Clear aligners were the most preferred treatment option, chosen by 62 (52%) participants across both groups, while cosmetic improvement was the primary motivation for 55 (~47%) participants. Time, cost, and appearance during treatment were the main obstacles. Despite these concerns, 34 (66.7%) younger participants and 42 (62.7%) older participants expressed interest in treatment, with a high willingness to invest, particularly among 43 (84.4%) younger participants.

Conclusions

Awareness and motivation for orthodontic treatment among adults are increasing, with younger adults showing greater knowledge and willingness to invest. Cosmetic enhancement remains the leading motivator, while time, cost, and aesthetic concerns during treatment are major barriers. Tailored educational strategies addressing age-related perceptions can encourage more adults to pursue orthodontic care, ultimately improving dental health, aesthetics, and self-confidence.

## Introduction

For decades, orthodontic treatment was widely believed to be most effective when performed during childhood or adolescence, capitalizing on the significant growth potential during these developmental stages [1]. This approach aimed to guide craniofacial development effectively by leveraging natural growth to align teeth and correct skeletal discrepancies [2]. Early intervention was emphasized not only to achieve more predictable and stable outcomes but also to prevent the progression of severe malocclusions, thereby reducing the complexity and duration of future treatments. Consequently, orthodontic care historically focused on younger patients during periods of active growth.

In contrast, adults historically avoided orthodontic treatment due to several factors. Limited technology and the lack of suitable appliances for non-growing individuals made treatment less effective in addressing complex dental and skeletal issues [3]. Social stigma and aesthetic concerns, particularly regarding the visibility of traditional metal braces, also discouraged many adults from seeking treatment [4]. Moreover, a lack of awareness about the effectiveness of adult orthodontics, coupled with the misconception that treatment was appropriate only for children, further contributed to this trend [5]. Biological factors such as slower bone remodeling and reduced tissue adaptability in adults also made tooth movement more difficult, often prolonging treatment duration [6]. Financial constraints and competing life priorities frequently led adults to defer orthodontic care [7]. These factors collectively contributed to the underrepresentation of adults in orthodontic treatment.

The development of aesthetic brackets has significantly increased the acceptance of orthodontic treatment among adults by addressing concerns about visibility and comfort. Ceramic brackets, introduced in the 1980s, provided a less noticeable alternative to metal braces [8], followed by composite and polycarbonate brackets, which offered improved aesthetics and durability [9]. Self-ligating aesthetic brackets further enhanced comfort and reduced friction, while lingual braces, placed on the inner surface of the teeth, provided complete invisibility [10]. The advent of clear aligners, such as Invisalign, revolutionized adult orthodontics by offering virtually invisible, removable appliances [11,12]. These advancements have motivated many adults to seek treatment for improved dental health, smile aesthetics, and self-confidence. Increased accessibility, ongoing technological innovations, and the influence of social media have further contributed to the growing popularity of adult orthodontic care.

Beyond these motivations, various demographic and psychosocial factors, such as age, gender, and socioeconomic status, influence the decision to pursue treatment. Adults often have different motivations and constraints compared to younger patients, and those from higher socioeconomic backgrounds are more likely to have access to orthodontic care [13,14]. Understanding patients’ expectations and aligning them with realistic therapeutic outcomes enhances satisfaction with treatment results.

Despite these advancements, limited information is available in the literature regarding adult awareness of the potential benefits of orthodontic treatment, leading to hesitancy and delayed decision-making. Understanding the relationship between awareness, motivation, and willingness is essential to identify barriers that prevent adults from seeking orthodontic care. Therefore, this study aimed to assess the level of awareness about modern orthodontic options, explore the motivational factors influencing adult patients, and evaluate their willingness to undergo treatment. The findings will provide valuable insights for developing targeted educational strategies and improving outreach efforts, ultimately promoting better oral health outcomes and increasing the acceptance of orthodontic treatment among adults.

## Materials and methods

This study employed a quantitative, cross-sectional design to examine adult perceptions of age as a barrier to orthodontic treatment. The structured questionnaire developed for this purpose underwent rigorous content validation by a panel of five experts, who rated each item on a 4-point relevance scale. Items rated as highly relevant (scores of 3 or 4) by at least three experts were retained, and the Item-Level Content Validity Index was calculated to confirm the adequacy of the questionnaire.

A total of 118 adult participants completed the validated questionnaire, which was distributed online via Google Forms (Google LLC, Mountain View, CA, USA). Participants were recruited based on their willingness to participate and were divided into two age groups: below 40 years and above 40 years. A convenience sampling technique, a non-probability method, was used to facilitate efficient data collection from a defined population. Data were collected at a single point in time using this validated instrument.

Following data collection, responses were exported from Google Forms into Microsoft Excel (Microsoft Corporation, Redmond, WA, USA) for systematic data cleaning, which included identifying and correcting inconsistencies, missing values, and entry errors. The cleaned dataset was then coded and imported into IBM SPSS Statistics for Windows, Version 21.0 (Released 2012; IBM Corp., Armonk, NY, USA) for statistical analysis. Descriptive statistics were used to summarize participant demographics and response distributions, while inferential statistics, specifically the chi-square test, were applied to examine associations between categorical variables. The threshold for statistical significance was set at p < 0.05.

Ethical approval for the study was obtained from the Ethical Committee of Sri Rajiv Gandhi College of Dental Sciences and Hospital (approval SRGCDS/2025/104), ensuring adherence to ethical standards for data collection, confidentiality, and participant consent.

## Results

**Figure 1:**
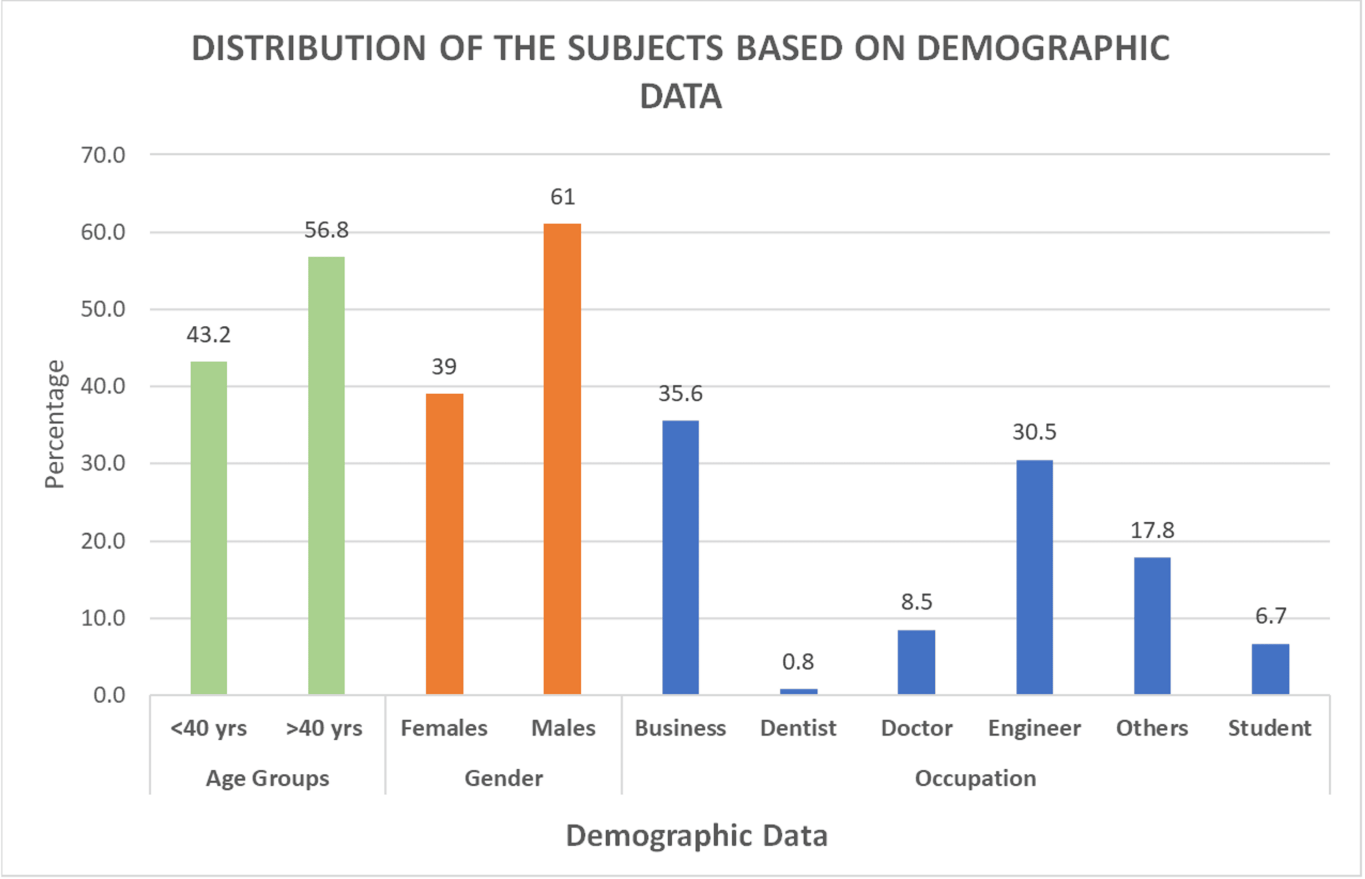
Representation of demographic data

A total of 118 participants took part in the study. Among them, 83 (56.8%) participants were over 40 years old, while 63 (43.2%) were under 40. Males comprised 89 (61%) of the sample, and females comprised 57 (39%). The majority of participants were business professionals (52, 35.6%), followed by engineers (45, 30.5%), doctors (12, 8.5%), students (10, 6.7%), and others (26, 17.8%) (Table 1, Figure 1).

**Table 1:** Distribution of participants based on age, gender, and occupation

Category	Variable	Frequency (n)	Percentage (%)
Age group	<40 years	63	43.2
	>40 years	83	56.8
Gender	Female	57	39
	Male	89	61
Occupation	Business	52	35.6
	Dentist	1	0.8
	Doctor	12	8.5
	Engineer	45	30.5
	Others	26	17.8
	Student	10	6.7

**Figure 2:**
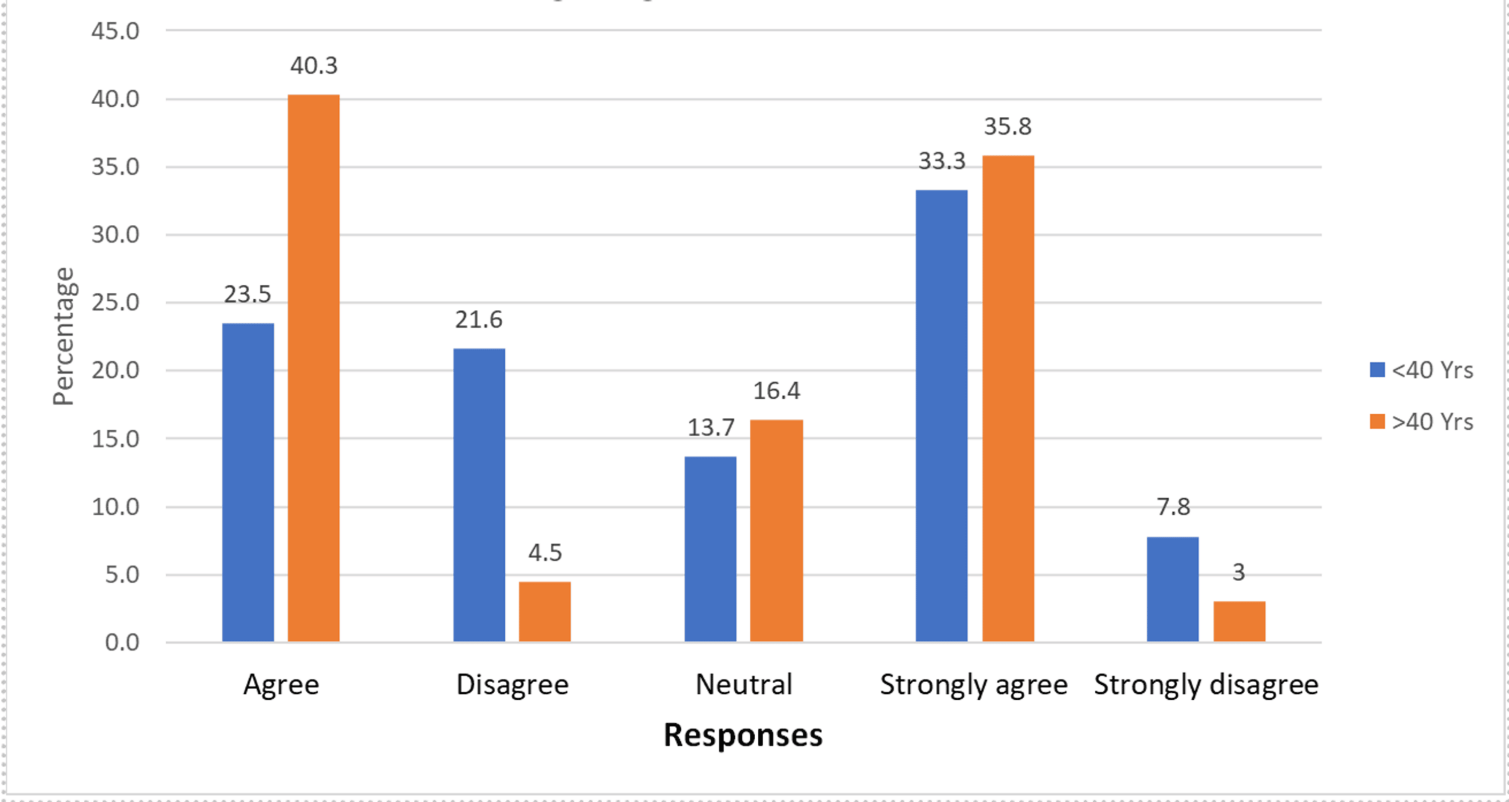
Perceptions of age as a barrier to orthodontic treatment

Participants under 40 were more aware of orthodontists, with 49 (96.1%) recognizing them compared to 57 (85.1%) among those over 40. Only a small percentage in both age groups were unaware of orthodontists. Older adults were more likely to perceive age as a barrier, with 51 (76.1%) participants over 40 agreeing with this statement, compared with 29 (56.8%) participants under 40. This difference was statistically significant (Figure 2).

Most participants, 36 (70.6%) under 40 and 53 (79.1%) over 40, considered adult orthodontic treatment common. More than half believed adults face greater challenges during treatment. Awareness of the benefits of adult orthodontic treatment was high in both groups: 40 (78.4%) under 40 and 48 (71.6%) over 40 were informed about these benefits. Similar levels of knowledge were observed regarding the functional advantages of treatment.

Many respondents recognized the importance of adult orthodontic treatment. Among them, 30 (58.8%) under 40 and 32 (47.8%) over 40 emphasized its cosmetic and functional value. Consultations with dentists or orthodontists were the primary source of information, with 23 (45.1%) participants under 40 and 40 (59.7%) participants over 40 relying on this source, while online platforms and social media played a lesser role.

**Figure 3:**
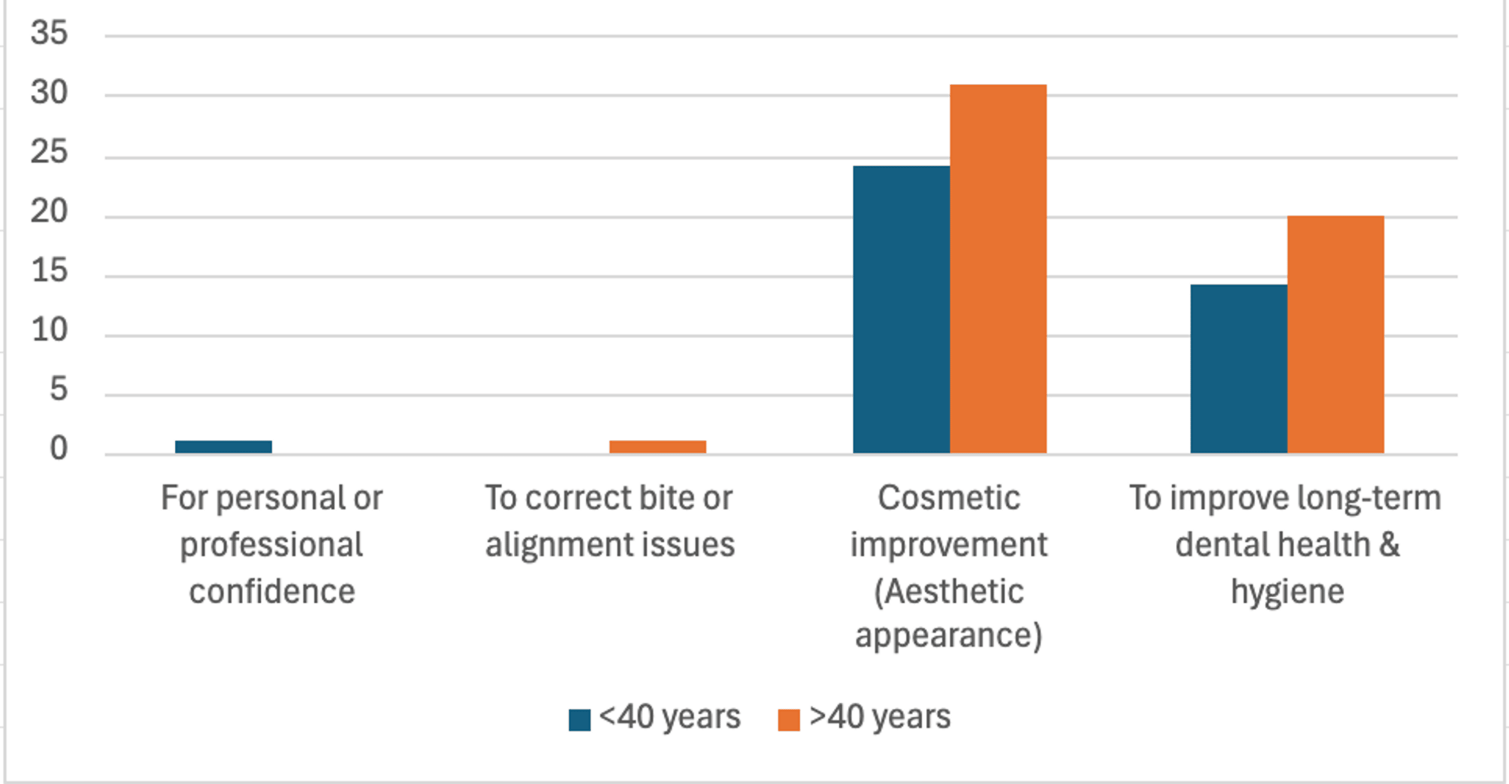
Motivation for seeking orthodontic treatment as adults

Clear aligners were the most preferred treatment option, with 62 (52%) participants across both age groups favoring them. Ceramic or clear braces were more preferred among participants over 40 (16, 23.9%). Cosmetic improvement was the main motivation for seeking treatment, cited by approximately 55 (47%) participants overall. Improving dental health was the next most common reason, mentioned by 14 (28%) under 40 and 20 (30%) over 40 (Figure 3).

Most participants believed that aligned teeth improve facial appearance, with 31 (60.8%) participants under 40 and 45 (67.2%) participants over 40 agreeing. Around 48 (40%) participants across both groups expressed concerns about braces affecting their appearance or social life. Approximately 70 (59%) participants indicated they would recommend orthodontic treatment to peers in their age group. Time and cost were identified as the major concerns, cited by 43 (49.2%) participants in both groups.

The primary reason for not seeking treatment earlier was the belief that it was unnecessary, with 28 (54.9%) participants under 40 and 36 (53.7%) participants over 40 expressing this view. A higher percentage of older adults (6, 9%) believed it was too late for them to undergo treatment.

**Figure 4:**
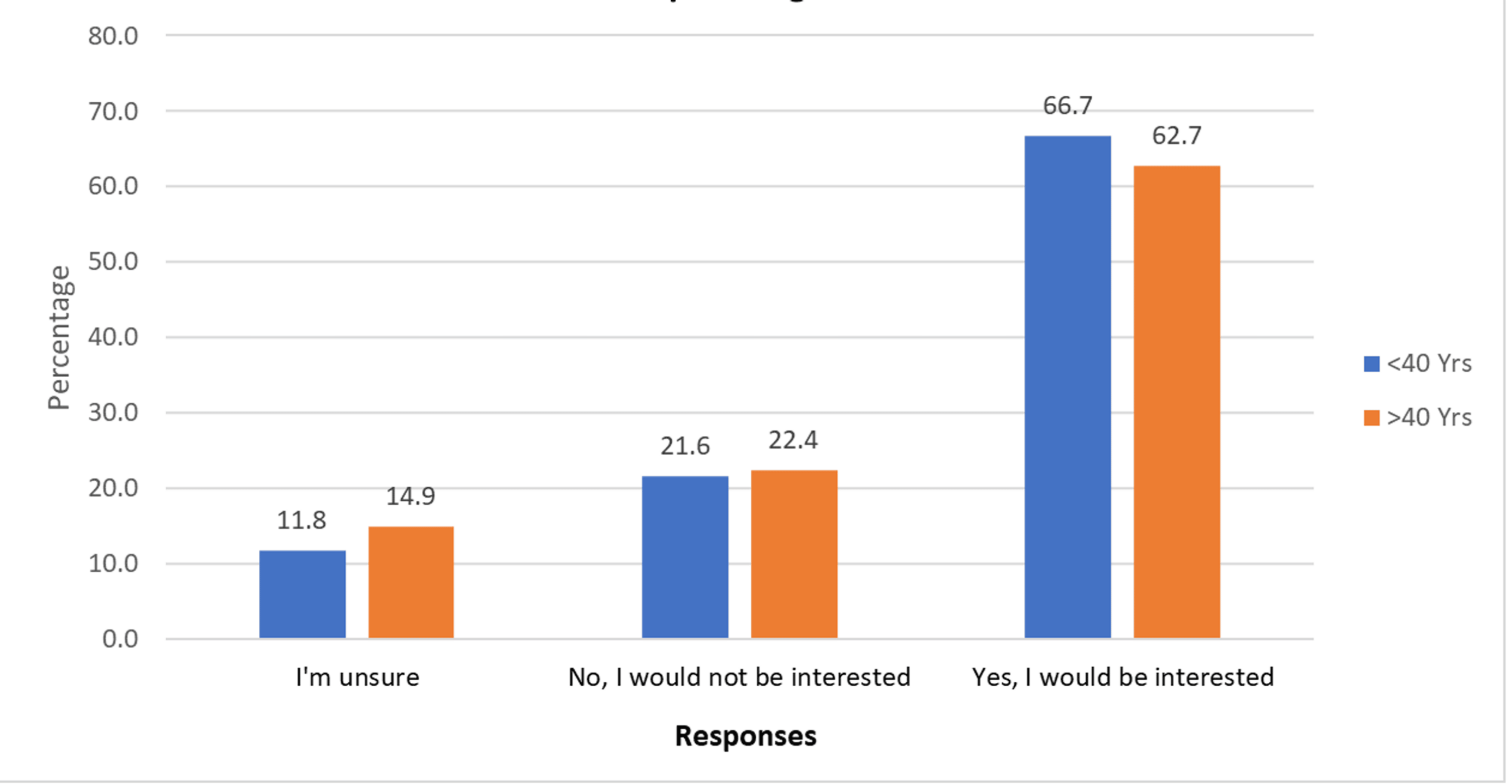
Willingness to undergo orthodontic treatment as an adult

Interest in treatment during adulthood remained strong, with 34 (66.7%) under 40 and 42 (62.7%) over 40 expressing enthusiasm (Figure 4). Most participants believed orthodontic treatment would enhance their self-confidence. Willingness to invest in treatment was also high, particularly among those under 40, with 43 (84.4%) indicating a strong intention to prioritize undergoing treatment.

## Discussion

Understanding why adult patients choose orthodontic treatment and how it affects their quality of life is important, as adults face unique challenges in orthodontic care. Their decisions are often influenced not only by functional needs but also by aesthetic concerns, self-esteem, and social acceptance. Evidence also shows that orthodontic treatment can significantly improve adults’ oral health-related quality of life, underscoring the importance of exploring their motivations and expectations [15].

A recent study by the Oral Health Foundation’s (formerly known as the British Dental Health Foundation) National Dental Survey found that one in two adults nearing middle age would consider orthodontic treatment to improve their smile. Among all age groups, adults aged 36-45 were the most likely to seek cosmetic dental treatment to enhance the appearance of their teeth [16]. Similarly, data from the Dental Practice Board reflected this trend, showing a 250% increase in orthodontic claims between 1992 and 2004, highlighting the expanding role of orthodontics in modern dental care. Additionally, members of the British Orthodontic Society (BOS) reported that adults accounted for approximately 17% of the average orthodontist’s workload, with most patients falling into the 18-30 age group [17]. Keim et al. also reported that the median percentage of adult cases increased from 15.4% in 1981 to 20% in 2007 [18]. Furthermore, Sandhya et al. found that adults constituted approximately 51.3% of orthodontic patients, surpassing the number of children and adolescents undergoing treatment [19]. These findings indicate that adults are increasingly aware of and motivated to seek orthodontic treatment, primarily for cosmetic reasons.

The present study aimed to assess participants’ awareness, motivation, and willingness to pursue orthodontic treatment, exploring whether age is perceived as a barrier and comparing responses between those under and over 40 years of age. In this study, 49 (96.1%) participants under 40 years and 57 (85.1%) participants over 40 were aware that orthodontists specialize in aligning teeth, indicating a high level of awareness in both groups. This aligns with the findings of Arslan and Elekdag-Türk, who reported that over 85% of respondents knew that an orthodontic specialist provides orthodontic treatment, while only 7% were unaware of orthodontics as a specialty and believed that general dentists could align teeth [20].

A statistically significant difference was observed between the two age groups regarding agreement with the statement “Age is a barrier to getting orthodontic treatment.” Among participants over 40 years, 27 (40.3%) agreed and 24 (35.8%) strongly agreed, suggesting that older individuals were more likely to perceive age as a significant barrier to treatment. This finding is consistent with the study by Kim, which showed that 48.5% of adults had a positive perception of orthodontic treatment, with those in their 20s (63.2%) showing the highest positivity, while those in their 40s and 50s had lower rates (46.2% and 45.1%, respectively), often believing they were too old for treatment [21]. Similarly, Pattanaik et al. reported that age impacts patient compliance, with younger patients exhibiting higher adaptability and compliance compared to older adults, who may find it harder to adjust to orthodontic care requirements [15].

When motivations for seeking orthodontic treatment were assessed, cosmetic improvement emerged as the primary motivator for both age groups (24 (47.1%) under 40 years and 31 (46.3%) over 40 years), followed by improving long-term dental health (27.5% and 29.9%, respectively). These findings align with the study by Amuasi and Owusu-Ansah, who reported that common motivations included aesthetic concerns (38.2%), correction of misaligned teeth (28.1%), and dentist recommendations (19.2%) [22]. Likewise, Pabri et al. found that the main motivations for adults seeking orthodontic treatment were to straighten teeth and improve their smile, followed by improving bite and function [23].

When willingness to undergo orthodontic treatment was assessed, most respondents expressed interest in pursuing treatment, with 34 (66.7%) participants under 40 and 42 (62.7%) participants over 40, indicating similar levels of interest across both groups. According to a survey by the American Association of Orthodontists, the number of adult orthodontic patients has steadily increased, from an average of 129 in 2012 to 178 in 2018, reflecting a 27.53% rise over six years [24]. Similarly, a recent BOS survey reported that more than 76% of orthodontists observed an increase in adult patients within the past three years, with 83% of these patients aged 26-55 years [25].

These findings highlight the evolving landscape of orthodontic care, with adult treatment gaining wider acceptance. The persistence of awareness and motivation across age groups reflects a societal shift in attitudes toward appearance and oral health. The sustained interest in orthodontic treatment among adults underscores growing recognition of its long-term functional and aesthetic benefits.

However, this study has several limitations. The relatively small and uneven sample size may reduce statistical power and limit the generalizability of results. The reliance on self-reported data introduces potential biases such as social desirability and recall errors, which may affect validity. The use of basic statistical analyses, such as the chi-square test, restricts exploration of more complex relationships. Furthermore, potential response bias may have influenced participant answers. Therefore, caution should be exercised when interpreting the findings, and future research with larger, more balanced samples and robust analytical methods is recommended.

## Conclusions

This study reveals that adults today are generally open to undergoing orthodontic treatment, with younger adults showing greater awareness of what orthodontists do and more willingness to invest in their dental care. People of all ages recognize the value of orthodontics not only for enhancing their smiles but also for improving overall dental health. However, older adults sometimes worry that age may make treatment more difficult. Clear aligners are the most preferred option, primarily due to their aesthetic appeal. The main obstacles identified include treatment duration, cost, and concerns about appearance while wearing braces. Overall, most adults view orthodontic treatment positively, believing it can boost both confidence and oral health. By promoting awareness and providing age-appropriate guidance, more adults can overcome doubts, especially the perception that they are too old for treatment, and feel encouraged to pursue orthodontic care.

## Appendices

**Table 2:** Survey questionnaire form

Impact of awareness, motivation, and willingness on perceptions of age as a barrier in adult orthodontic treatment
1. What is your age?
Mark only one oval.
<40
>40
2. What is your gender?
Mark only one oval.
Male
Female
3. What is your occupation?
Mark only one oval.
Doctor
Engineer student others
Business
4. Are you aware that orthodontists specialize in aligning teeth?
Mark only one oval.
Yes, I'm aware
No, I was not aware. I'm not sure
5. How strongly do you agree with the statement: “Age is a barrier to getting orthodontic treatment”?
Mark only one oval.
Strongly agree
Agree Neutral Disagree
Strongly disagree
6. In your opinion, how prevalent is orthodontic treatment among adults?
Mark only one oval.
Very prevalent
Somewhat prevalent Not very prevalent Not prevalent at all
I'm not sure
7. Why did you not undergo orthodontic treatment previously?
Mark only one oval.
I didn't think I had dental issues that required treatment
I couldn't afford the cost
I was not aware of the options for adults
The inconvenience (time and lifestyle changes) was a concern I didn't want to wear traditional braces
I felt it was too late to start treatment as an adult
I wasn't motivated to pursue it at that time
8. If you had never received orthodontic treatment, would you be interested in pursuing it now as an adult?
Mark only one oval.
Yes, I would be interested
No, I would not be interested I'm unsure
9. Do you believe that adults experience more discomfort or challenges during orthodontic treatment than younger patients?
Mark only one oval.
Yes, adults likely experience more discomfort/challenges
No, I don't think adults face more diﬃculty I'm unsure
10. How aware are you of the beneﬁts of orthodontic treatment for adults?
Mark only one oval.
Very aware
Somewhat aware Not very aware Not aware at all
I'm not sure
11. Which of the following have you used to learn about orthodontic treatment in adults?
Mark only one oval.
Orthodontist/dentist consultations/Health professionals
Online articles and websites Social media
Advice from friends and family
I have not sought any information
12. If you've not had treatment, what type of treatment would you consider now?
Mark only one oval.
Clear aligners
Ceramic or clear braces Traditional metal braces
Lingual braces (braces on the inner side of teeth)
I would not consider orthodontic treatment
13. How much do you know about the functional beneﬁts of orthodontic treatment (e.g., improved bite, easier cleaning, better function or overall dental health)?
Mark only one oval.
I know a lot about the functional beneﬁts
I know a moderate amount I know a little
I don't know much about the functional beneﬁts
I don't know anything about the functional beneﬁts
14. What would be your primary motivation for seeking orthodontic treatment as an adult?
Mark only one oval.
Cosmetic improvement (Aesthetic appearance)
To correct bite or alignment issues
To improve long term dental health and hygiene For personal or professional conﬁdence
15. Do you think properly positioned teeth contribute to better facial appearance?
Mark only one oval.
Yes, they signiﬁcantly improve facial appearance
Yes, but only to a small extent
No, I don't think teeth position affects facial appearance I'm not sure
16. If you were to align your teeth, do you think it would improve your self conﬁdence?
Mark only one oval.
Yes, I believe it would signiﬁcantly boost my conﬁdence
Yes, I think it would give me some boost in conﬁdence No, I don't think it would affect my conﬁdence much
No, I don't believe it would have any impact on my conﬁdence
I'm unsure
17. If you could improve your smile with orthodontic treatment, how willing would you be to invest in it?
Mark only one oval.
Very willing - I would prioritize it
Somewhat willing - I would consider it if it ﬁts my budget
Not very willing - I would need to be convinced its worth the investment
Not willing at all - I don't think I would invest in orthodontic treatment I'm unsure
18. Do you have concerns about the impact braces could have on your appearance or social life?
Mark only one oval.
Yes, I am worried it might negatively impact my social life or appearance
Yes, but the concerns are not signiﬁcant
No, I don't think braces will affect my appearance or social life I'm unsure
19. If you've undergone treatment as an adult, would you advice others in your age group to do the same?
Mark only one oval.
Yes, I would encourage other adults to consider it
Yes, but I would caution them about the challenges or costs No, I would not encourage others to pursue it
I'm unsure
20. How likely are you to pursue orthodontic treatment if clear aligners were an option instead of traditional braces?
Mark only one oval.
Very likely - I would deﬁnitely consider it
Likely - I'm not sure I would choose it
Very unlikely - I would prefer other options or not pursue treatment at all I'm unsure
21. What concerns do you have about undergoing orthodontic treatment as an adult?
Mark only one oval.
The time commitment required
The cost of treatment
Concerns about discomfort or pain I have no concerns
22. How important do you think it is for adults to consider orthodontic treatment for both cosmetic and health-related reasons?
Mark only one oval.
Very important - I believe it is valuable for adults
Somewhat important - It's helpful, but not a priority for everyone Not important - I don't think adults need orthodontic treatment I'm unsure
